# Dual effects of single-walled carbon nanotubes coupled with near-infrared radiation on *Bacillus anthracis* spores: inactivates spores and stimulates the germination of surviving spores

**DOI:** 10.1186/1754-1611-7-19

**Published:** 2013-08-21

**Authors:** Xiuli Dong, Yongan Tang, Marvin Wu, Branislav Vlahovic, Liju Yang

**Affiliations:** 1 Biomanufacturing Research Institute and Technology Enterprise (BRITE) and Department of Pharmaceutical Sciences, North Carolina Central University, Durham, NC 27707, USA; 2 Department of Mathematics and Physics, North Carolina Central University, Durham, NC 27707, USA

**Keywords:** Single walled carbon nanotubes, *Bacillus anthracis* spores, Near infrared radiation, Germination, Dipicolinic acid, Gene expression

## Abstract

**Background:**

*Bacillus anthracis* is a pathogen that causes life-threatening disease--anthrax. *B. anthracis* spores are highly resistant to extreme temperatures and harsh chemicals. Inactivation of *B. anthracis* spores is important to ensure the environmental safety and public health. The 2001 bioterrorism attack involving anthrax spores has brought acute public attention and triggered extensive research on inactivation of *B. anthracis* spores. Single-walled carbon nanotubes (SWCNTs) as a class of emerging nanomaterial have been reported as a strong antimicrobial agent. In addition, continuous near infrared (NIR) radiation on SWCNTs induces excessive local heating which can enhance SWCNTs’ antimicrobial effect. In this study, we investigated the effects of SWCNTs coupled with NIR treatment on *Bacillus anthracis* spores.

**Results and discussion:**

The results showed that the treatment of 10 μg/mL SWCNTs coupled with 20 min NIR significantly improved the antimicrobial effect by doubling the percentage of viable spore number reduction compared with SWCNTs alone treatment (88% vs. 42%). At the same time, SWCNTs-NIR treatment activated the germination of surviving spores and their dipicolinic acid (DPA) release during germination. The results suggested the dual effect of SWCNTs-NIR treatment on *B. anthracis* spores: enhanced the sporicidal effect and stimulated the germination of surviving spores. Molecular level examination showed that SWCNTs-NIR increased the expression levels (>2-fold) in 3 out of 6 germination related genes tested in this study, which was correlated to the activated germination and DPA release. SWCNTs-NIR treatment either induced or inhibited the expression of 3 regulatory genes detected in this study. When the NIR treatment time was 5 or 25 min, there were 3 out of 7 virulence related genes that showed significant decrease on expression levels (>2 fold decrease).

**Conclusions:**

The results of this study demonstrated the dual effect of SWCNTs-NIR treatment on *B. anthracis* spores, which enhanced the sporicidal effect and stimulated the germination of surviving spores. SWCNTs-NIR treatment also altered the expression of germination, regulatory, and virulence-related genes in *B. anthracis.*

## Background

*Bacillus anthracis* is a pathogen that causes life-threatening disease--anthrax. *B. anthracis* spores are highly resistant to extreme temperatures and harsh chemicals. The dormancy of spores can remain decades or even centuries until the opportunity for infection arises. Animals can be infected by spores through inhalation and grazing. Humans may develop cutaneous anthrax through the exposure of skin to infected animals, or ingestion anthrax through eating contaminated food, or inhalation anthrax by breathing in the spores or materials contaminated with the spores.

Inactivation of *B. anthracis* spores is important to ensure the environmental safety and public health. The 2001 bioterrorism attack involving anthrax spores have brought acute public attention and triggered extensive research on inactivation of *B. anthracis* spores. Common antimicrobial agents including bleach, chlorine dioxide, hydrogen peroxide, paraformaldehyde, were used in federal decontamination responses to the bioterrorism attack [1]. Whitney et al. reviewed much of the available literature on the inactivation of *Bacillus* spores that is relevant to the inactivation of *B. anthracis*[2]. However, because the efficiencies of many of these methods were determined using *Bacillus* species other than *B. anthracis,* a number of methods and reagents that reportedly killed *B. anthracis* spores suffered from low efficacy [2]. The high lethality, resistivity, aerosolization ability, and the potential to be a bioterrorism agent, have heightened the research interest in the development of new sporicidal agents/methods for inactivating *B. anthracis* spores.

Single-walled carbon nanotubes (SWCNTs) as a class of emerging nanomaterial have been reported as a strong antimicrobial agent. Kang et al. reported the first direct evidence of SWCNTs’ strong antimicrobial activity to bacterial cells [3]. Since then, a number of studies have been reported on the antimicrobial effects of carbon nanotubes (CNTs) and the following related influence factors, including CNT diameter [4], length [5], aggregation [6], concentration [6,7], CNTs’ surface groups, buffer solution, and bacterial species [7], contact time and intensity [6-8]. Dong et al. reported that SWCNTs might be an effective alternative to antibiotics in dealing with drug-resistant and multidrug-resistant bacterial strains because of the physical mode of bactericidal action that SWCNTs display [9]. Our most recent study showed that SWCNTs exhibited antimicrobial activity to *B. anthracis* vegetative cells at the similar level to other bacterial species [10]. The mechanisms of SWCNT’s antimicrobial activity to bacterial cells depended essentially on the direct contact resulting in cell membrane damage [3,5,7], but also included oxidative stresses due to direct oxidation of cellular components, and secondary oxidation of cellular lipid bilayer by reactive oxygen species [4,11-14].

However, only a limited number of studies have been published regarding the interactions between SWCNTs and bacterial spores. Spores are structurally different from vegetative cells. *B. anthracis* spores are encased in a thick multilayered coat, which is further surrounded by the exosporium. The exosporium is composed of a paracrystalline basal layer with a hexagonal lattice structure and a hair-like outer region [15]. Monosaccharide -functionalized SWCNTs interacted with *B. anthracis* spores with the mediation of a divalent cation such as Ca^2+^ to form significant spore aggregation [16]. SWCNTs showed high adsorption affinity towards *B. subtilis* spores [17]. The use of aggregation formed between SWCNTs and spores was investigated for the removal of *Bacillus* spores from environment [17]. Notably, these studies did not mention the antimicrobial effect of SWCNTs on *Bacillus* spores. Our recent studies have initiated the study of SWCNTs’ action on *B. anthracis* spores and found that SWCNTs did not exhibit antimicrobial effect to bacterial spores in deionized water suspensions at concentrations less than 150 μg/mL [10]. Considering the protective structure of bacterial spores and the mechanisms of SWCNTs inactivating bacterial cells, it is not surprising that SWCNTs showed little antimicrobial effect to *B. anthracis* spores.

Fortunately, besides its intrinsic antimicrobial activity, SWCNTs possess other properties that can be used in combination to enhance its antimicrobial activity. For example, SWCNTs show strong absorbance to 700- to 1100-nm NIR light and then convert it into heat, while biological systems are transparent at this wavelength range [18]. Continuous NIR radiation on SWCNT-internalized cells can cause cell death because of excessive local heating to SWCNTs [19]. Recent results with bacterial cells (*Salmonella*) showed that SWCNTs-NIR treatment resulted in a 3-fold decrease in viable cells compared to SWCNTs alone [20].

In this study, we investigated the antimicrobial effects of SWCNTs-NIR treatment on the inactivation of *B. anthracis* spores and the germination of surviving *B. anthracis* spores in comparison to SWCNTs alone, and examined the alterations at the molecular level upon the SWCNTs-NIR treatment to *B. anthracis* spores, including the transcriptional expression levels of germination-related genes, regulatory genes, and virulence related genes.

## Results and discussion

### The NIR absorbance characteristics of SWCNTs

**Figure 1:**
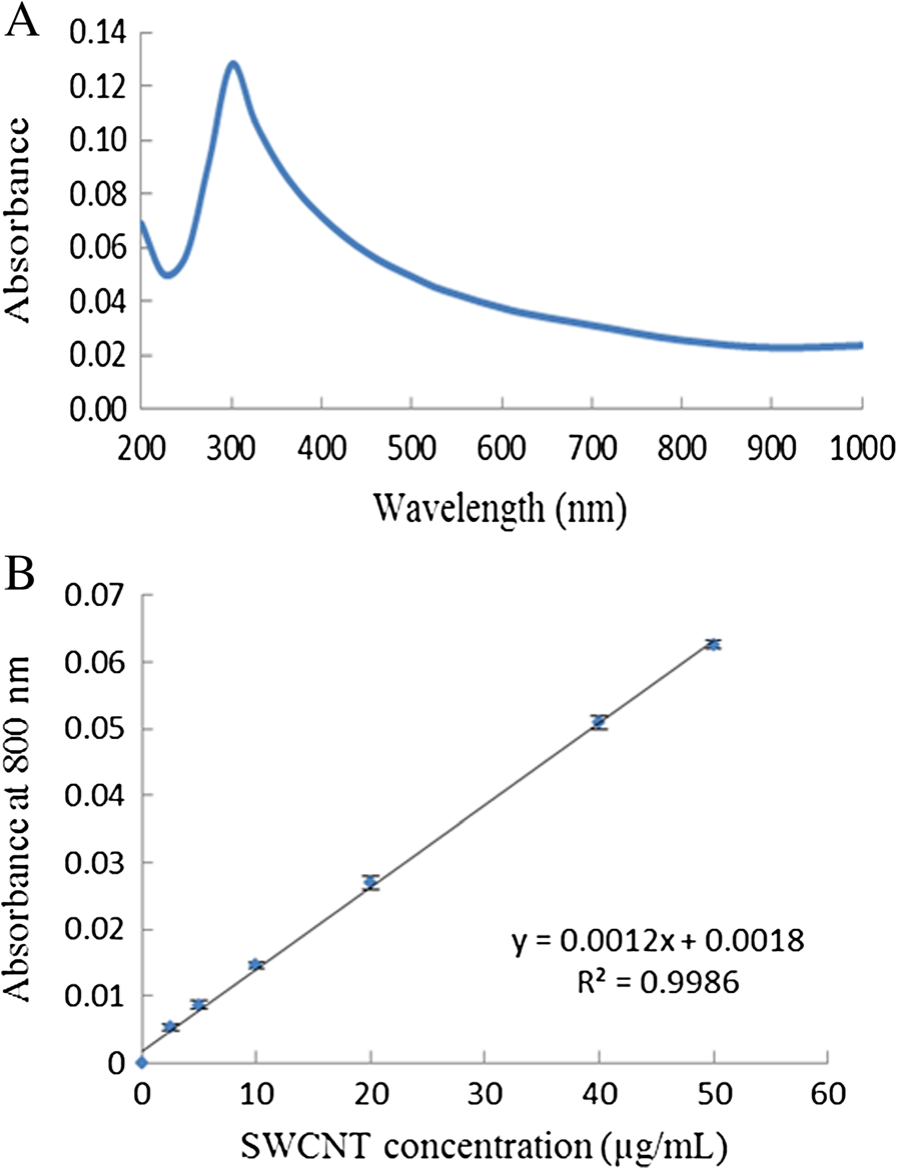
**The optical spectrum of SWCNTs solution. (A)** The UV-VIS-NIR spectrum of SWCNTs solution in DI-H_2_O at a concentration of 20 μg/mL. **(B)** The linear relationship between the absorbance at 800 nm and the concentration of SWCNTs. Error bars in **(B)** are standard deviations of triplicated experimental results.

We first examined the UV-VIS-NIR spectrum of SWCNTs, and Figure 1 shows the UV-VIS-NIR spectrum of 20 μg/mL SWCNTs in DI-H_2_O. The maximal absorption peak was at 300 nm, which corresponded to the plasmon absorption of SWCNTs. The maximal absorption wavelength of different batches of SWCNTs products varied slightly, likely because of the differences in SWCNTs’ composition, diameter, chiral index, chemical functionalization step, distribution of nanotube lengths, purification procedure, etc. In the NIR region (700 nm to 1000 nm), appreciable absorbance was observed. At 800 nm, a linear relationship between the optical density (OD) value and the SWCNTs concentration was represented by the regression equation of OD = 0.0012 C_SWCNTs_ + 0.0018 (R^2^ = 0.9986) (Figure 1B). This observation was consistent with previous studies [19,20].

### The effect of SWCNTs-NIR treatment on inactivation of *B. anthracis* spores

**Figure 2:**
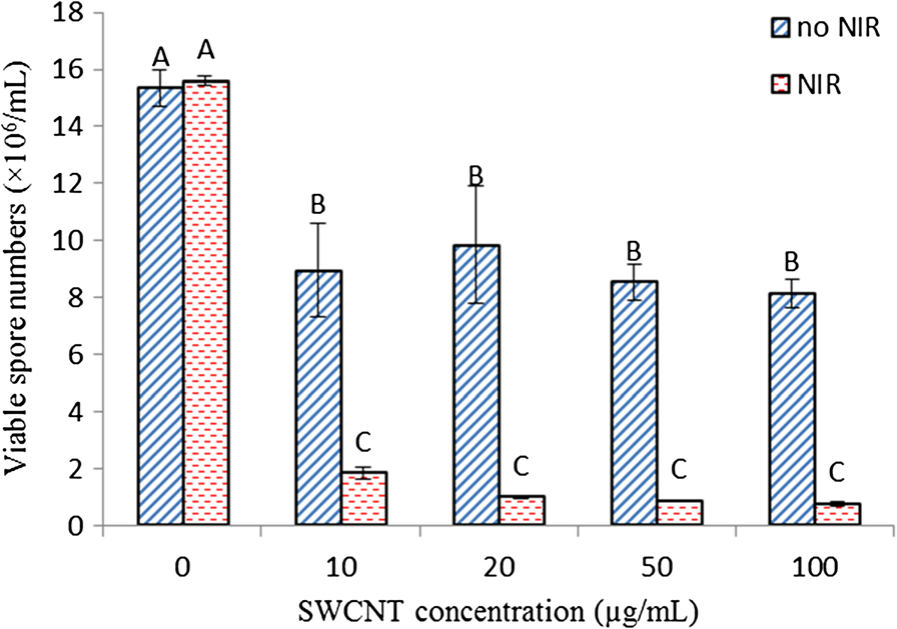
**Viable spore numbers after being treated with SWCNTs at various concentrations and coupled with or without 20-min NIR radiation.** Results are presented as the mean ± standard deviation. Different letters above the columns indicate statistically significant differences (P < 0.05).

The effect of SWCNTs-NIR treatment on *B. anthracis* spores was evaluated using the reduction of viable spores upon treatments determined by the traditional plating method. Figure 2 shows the reductions in viable spore number in the samples treated with SWCNTs alone and SWCNTs-NIR treatment. Compared to the SWCNTs alone treatment, the addition of SWCNTs (10-100 μg/mL) significantly reduced viable *B. anthracis* spore numbers from 1.53 × 10^7^ spores/mL to (8.15-9.85) × 10^6^ spores/mL (P < 0.05). However, the viable spore numbers did not decrease significantly with the increasing SWCNTs concentrations from 10 to 100 μg/mL(P > 0.05). With treatment of 100 μg/mL SWCNTs, the killing rate was about 46.9%, which was much lower than those of SWCNTs’ inactivation rates to *Bacillus* vegetative cells and other bacterial cells previously reported which ranged from 80% to >99% depending on different conditions [3,4,7]. The results indicate that SWCNTs alone are not as effective in killing spores as in killing vegetative bacterial cells. Considering the protective structure of spores, the result is not unexpected.

In contrast to SWCNTs alone, SWCNTs with NIR significantly reduced the viable spore numbers (P < 0.05) compared to the SWCNTs-treated samples and the untreated sample, while a 20 min-NIR treatment without SWCNTs displayed no effect on viable spore numbers (Figure 2). For example, using 10 μg/mL SWCNTs, the viable spore number decreased 42% without NIR and decreased 88% with co-treatment of NIR. When SWCNTs concentration was increased to 100 μg/mL, SWCNTs-NIR co-treatment reduced the viable spore number by 95%. The results clearly demonstrated that co-treatment of SWCNTs with NIR enhanced the spore inactivation compared to SWCNTs alone treatment, although SWCNTs-NIR treatment did not exhibit a strong dependence on SWCNTs’ concentration.

**Figure 3:**
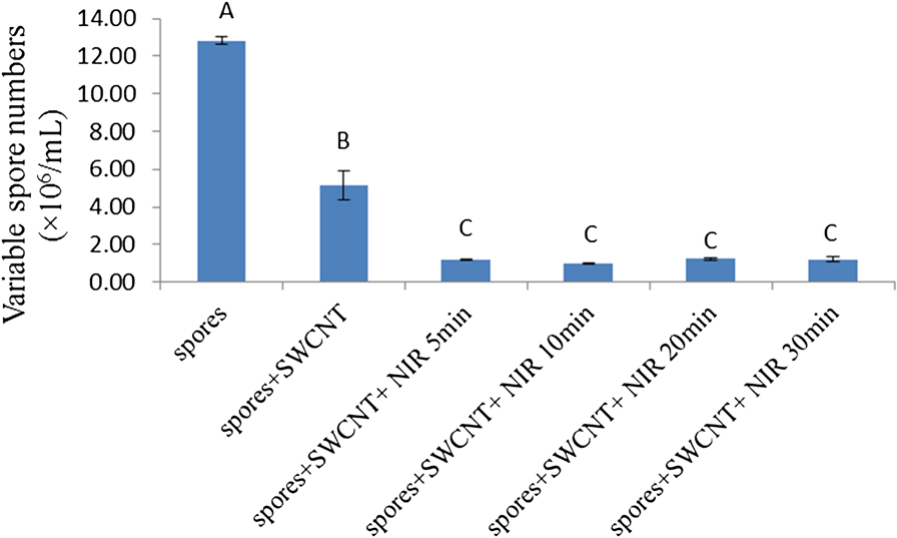
**Effects of the co-treatment of SWCNTs-NIR on the reduction of viable *B. anthracis *spore numbers.** Spores were treated with or without 20 μg/mL SWCNTs, and with or without NIR 5-30 min radiation. Results are presented as the mean ± standard deviation. Different letters above the bars indicate statistically significant differences (P < 0.05).

We also examined whether the duration of NIR treatment affected the efficiency of the co-treatment of SWCNTs-NIR. Figure 3 shows the reductions in viable spore numbers in the samples that received SWCNTs-NIR co-treatments with NIR treatment time varying from 5 to 30 min. Although the viable spore numbers in all the samples received SWCNTs-NIR co-treatment were significantly lower than that in the SWCNTs alone treated samples (P < 0.05), no significant difference was observed in the viable spore numbers among all the SWCNTs-NIR treated samples. The possible explanation of the observed results is that the effect of NIR reached its maximal level rapidly (within 5 min). Most likely, the effect of NIR on the enhancement of spore inactivation is the unique optical-thermal property of SWCNTs which rapidly convert NIR radiation into heat resulting in intensive local heating in spores, similar to the mechanism when it acted on cancer cells or bacterial cells [19,20]. We thus examined the temperature increase in the bulk solution. The temperature of 10 and 100 μg/mL SWCNTs suspensions increased from 23°C to a final temperature 25.4 and 26.6°C in 5 min, respectively. Although the temperature only slightly increased in the bulk suspensions, the temperatures on the SWCNTs attached to the spores could be much higher. Panchakesan et al. reported that localized heating of SWCNTs on exposure to 200 mW/cm^2^ of 800 nm NIR could lead to the explosions of SWCNT sheets in water due to the rapid temperature rise of the water molecules present in the nanopores between bundles to more than 100°C [21]. Explosions of loosely packed SWCNTs in air under laser treatment were reported by Ajayan et al. [22]. Further, a study has shown that carbon nanohorns (CNH), a family of carbon-based nanomaterials with similar atomic structure to CNTs, have the similar mechanism of NIR laser-triggered heating—optically stimulated electronic excitations being rapidly transferred to molecule vibration energies and causing local heating [23]. The heat is generated because of the high absorbance of SWCNTs in the NIR region (ϵ≈1.03 × 10^6^ M^-1^ cm^-1^ to 7.9 × 10^7^ M^-1^ cm^-1^), and such high absorbance is due to the electronic transitions between the first or second van Hove singularities of the nanotubes [19,24,25]. Heat is also generated in SWCNTs due to the electron-phonon coupling causing molecular vibrations [21]. The solution temperature reflected the extent of local heating of SWCNTs by NIR treatment. It was possible that the extreme local heat of SWCNTs in close contact with spores damaged some components of spores and thus enhanced the antimicrobial activity beyond the intrinsic antimicrobial activity of SWCNTs.

The results indicated that SWCNTs alone or SWCNTs-NIR treatment exhibited the antimicrobial activity on *B. anthracis* spores at the concentration 10 μg/ml SWCNTs, but their activity did not further increase with increasing concentration of SWCNTs from 10 μg/mL to 100 μg/mL. Previous results [26] using scanning electron microscopy (SEM) test showed that in this concentration range, spores were not saturated with SWCNTs in terms of surface coverage/interactions. In the SWCNTs-NIR treatment, the inactivation efficiency did not increase with the NIR treatment time beyond 5 min treatment time. Both observations were different from the observation on SWCNTs-NIR treatment to bacterial cells [20]. This may be due to the more protective structure of spores than bacterial cells and the limited capacity/function of SWCNTs and SWCNTs-NIR in the inactivation of spores. Further research will be necessary for more detailed mechanisms.

### Effect of SWCNTs-NIR treatment on spore germination

#### The growth of SWCNTs-NIR pretreated *B. anthracis* spores

**Figure 4:**
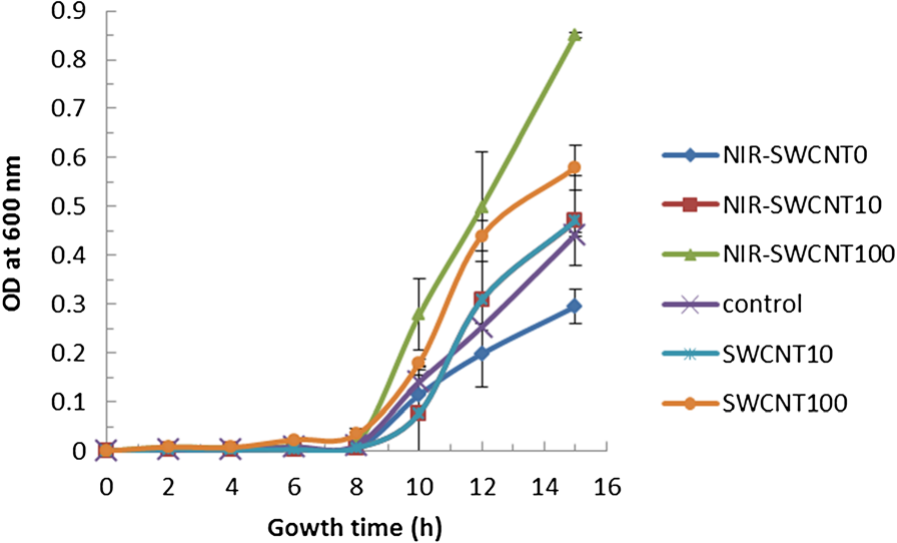
**Growth curves of *B. anthracis *spores in nutrient broth after they were treated with SWCNTs at various concentrations, coupled with or without 20-min NIR.** Error bars present the standard deviation of triplicated experimental results.

The growth of SWCNTs-NIR treated spores was examined by measuring optical density (OD) at 600 nm of the culture. Figure 4 shows the growth curves of *B. anthracis* spores in nutrient broth after the spores were pre-treated with SWCNTs (10 and 100 μg/mL) and SWCNTs with 20 min NIR treatments. The samples started exponential growth phases at similar suggesting that the treatments did not affect the lag phases, even though the viable spore number in these samples with different treatments were different (as shown in Figure 2). If the ODs at a given time during the exponential growth phase were compared, the samples treated with SWCNTs or SWCNTs-NIR showed slightly higher values than the control samples, indicating that both the SWCNTs treatment and SWCNTs-NIR treatment promoted the more rapid growth of surviving spores in nutrient broth, and the higher concentration of SWCNTs (100 μg/mL) was more effective at activating on growth. The possible reason for this growth activation in surviving spores was that partial physical damages on the spore walls by SWCNTs may trigger spore germination and local heating on spores by SWCNTs-NIR treatment. In *B. subtilis* spores, mechanical abrasion can trigger germination by activation of either of the spore’s cortex-lytic enzymes—CwlJ and SleB [27]. These enzymes are present in the spore in an active form but do not act until triggered by mechanical damage to spores or by germination stimuli, Ca^2+^-DPA for CwlJ or some other stimulus for SleB [27-29]. Since SWCNTs can partially damage the spore coat, making it more permeable [26], damage on the spores due to SWCNTs treatment may actually triggered spore germination and rapid growth. On the other hand, previous study demonstrated that brief heat treatment at a sublethal temperature on spores significantly increased the germination percentage of spores [30,31]. Under natural conditions, the activation of dormant spores in water occurred slowly, but a heat activation step most often potentiated germination of >90% of spore populations [30]. SWCNTs-NIR treatment caused local heat to spores, during which some surviving spores experienced sublethal heat and actually were stimulated to germinate by the heat. The stimulated germination of spores by SWCNTs-NIR was actually confirmed later by the observation of increased transcriptional expression levels of germination related genes. However, spores treated with NIR showed slightly lower ODs during the exponential phase compared to the control sample, even though NIR treatment did not reduce the viable spore compared to the control (as shown in Figure 2). The reason for this observation is not clear.

#### DPA release from *B. anthracis* spores treated with SWCNTs and SWCNTs-NIR

Spore germination is the first crucial step in the return of *B. anthracis* spore to vegetative growth leading to its pathogenicity. The germination process includes several steps: release of spore H^+^, release of dipicolinic acid (DPA) and its associate divalent cations, replacement of DPA by water, hydrolysis of the spore’s cortex, and swelling of the spore core [29]. DPA release is one of the early events that can be used to evaluate the germination of spores. To evaluate the effect of SWCNTs concentration on spore germination, the kinetics of relative fluorescence unit (RFU) changes based on DPA releases were monitored by the Tb-DPA method suitable for rapidly detecting spore germination immediately after treatment [32].

**Figure 5:**
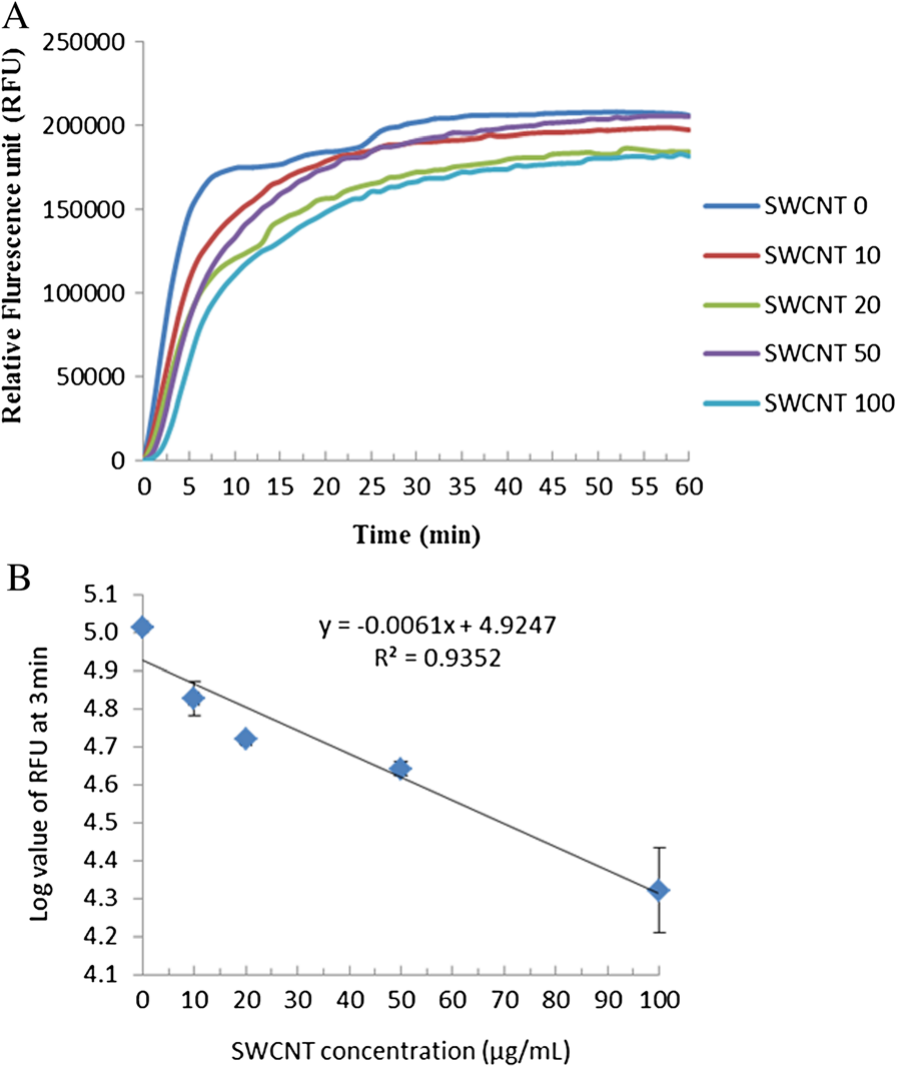
**The kinetics of DPA release from *B. anthracis *spores treated with SWCNTs. (A)** A group of curves of DPA release during the germination of *B. anthracis* spores after they were treated with different concentrations of single walled carbon nanotubes (SWCNTs). **(B)** The correlation between the log value of RFU in DPA measurement at 3 min of germination and the SWCNTs’ concentration. Error bars in **(B)** present the standard deviations of triplicated experimental results.

Figure 5A shows the kinetics of DPA release from the control and the SWCNTs-treated spore samples (4.5 × 10^7^ spores) during one hour germination. In all the samples, DPA contents quickly released in the first 5 min germination, and reached maximal DPA release after about 48 min germination. When comparing the maximal level of released DPA, the control released the highest concentration of DPA, while SWCNTs-treated samples released slightly lower concentrations of DPA. The DPA release of SWCNTs-treated samples did not display a clear SWCNTs concentration dependent trend. As it has been reported that the proportion of DPA released during germination was an indicator to test the percentage of viable spores capable of germination [33], the observed DPA release was consistent with the viable spore numbers in these treated samples which were not significantly different (as shown in Figure 2). When comparing the DPA release in the fast release period (within 5 min), the amount of DPA release at a given time showed a SWCNTs concentration-dependence. Figure 5B shows that the log value of RFU obtained from Tb-DPA method at 3 min of germination is correlated with SWCNTs concentration, following a linear equation Log RFU = -0.0061 C_SWCNTs_ + 4.9249 (*R*^*2*^ = 0.935, P < 0.05). This result indicated that SWCNTs treatment showed a concentration-dependent effect on the rate of DPA release during the early stage germination (within 5 min) of the surviving spores.

**Figure 6:**
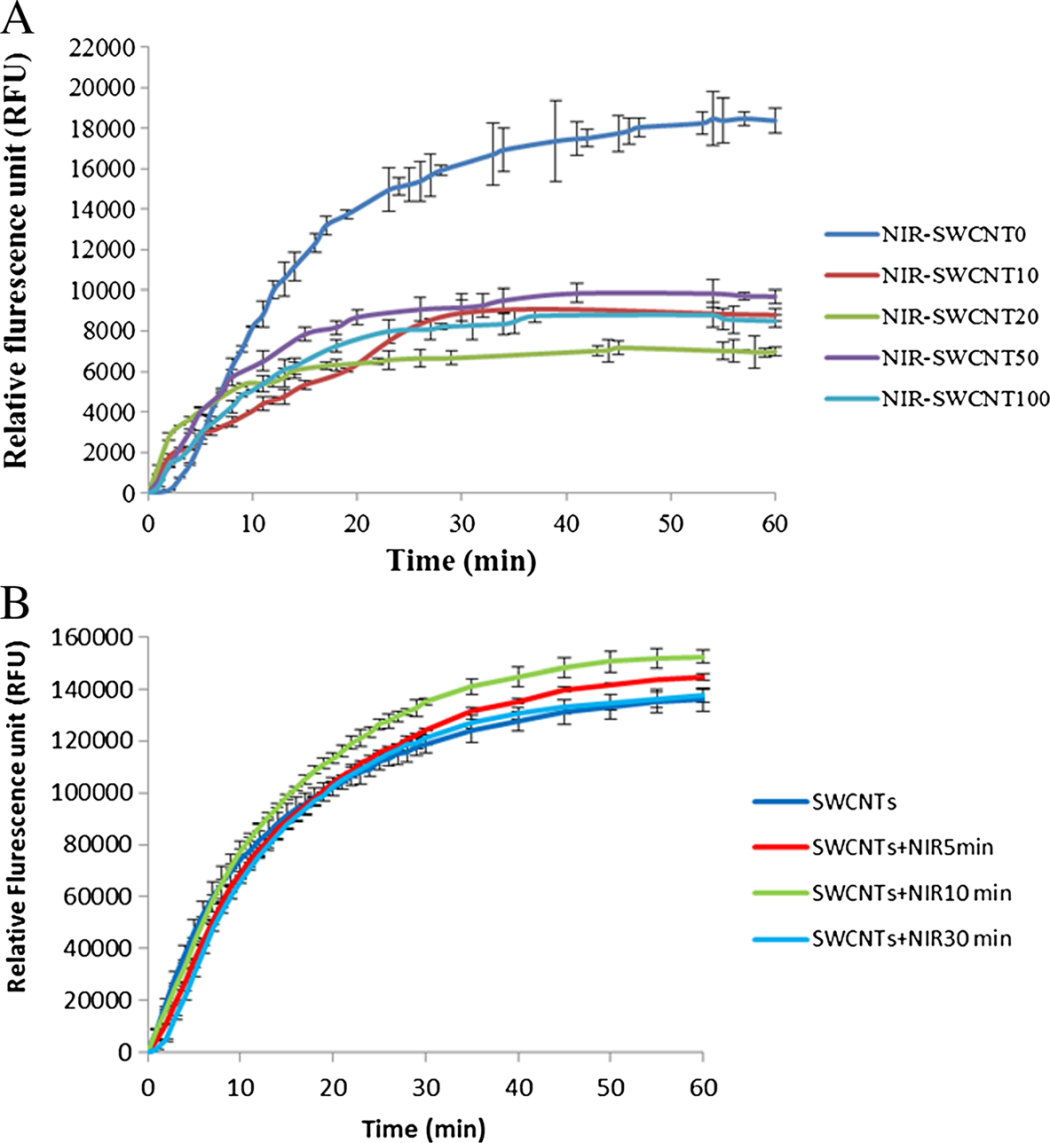
**The kinetics of DPA release from *B. anthracis *spores treated with SWCNTs-NIR. (A)** The kinetics of DPA release from *B. anthracis* spores that were treated with different concentrations of SWCNTs coupled with 20 min NIR radiation, during one hour of germination. Spore number: 5.3 × 10^6^ spores. **(B)** The kinetics of DPA release from *B. anthracis* spores that were treated with SWCNTs (20 μg/mL) and different time of NIR treatment during one hour of germination. Spore number: 3.6 × 10^7^ spores. Error bars present the standard deviations of triplicated experimental results.

Figure 6A shows the kinetics of DPA release from the spore samples (5.3 × 10^6^ spores) that received the co-treatment of SWCNTs with 20 min NIR during one hour germination. Kinetically, SWCNTs-NIR treated samples took 15-20 min to reach the maximal level of DPA release, which were slightly slower than the untreated control sample (5 min shown in Figure 5A). When the maximal level of released DPA was considered, the SWCNTs-NIR treated samples only reached about half of the DPA level released by the NIR-treated sample (Figure 6A). The results indicated that difference in the kinetics and the maximal level of DPA release between the control and the SWCNTs-NIR treated samples were correlated to the viable spore numbers in the control and treated samples. However, among the SWCNTs-NIR treated samples, no correlation between RFU of DPA and SWCNTs concentration was observed (for instance, at 3 min germination, R^2^ = 0.38, P < 0.05). This observation was different from samples treated with SWCNTs alone (as shown in Figure 5B), but was consistent with the viable spore numbers in SWCNTs-NIR treated samples (shown in Figure 2).

Figure 6B shows that kinetics of DPA release from *B. anthracis* spores (3.6 × 10^7^) treated with SWCNTs (20 μg/mL) coupled with different time periods of NIR treatment, in terms of the RFU vs. germination time. All the samples reached the maximal RFU at a similar time, indicating that the NIR treatment time did not affect the kinetics of DPA release from the spore samples. The maximal DPA levels released at 60 min of germination for all the SWCNTs-NIR treated samples were very close (RFU values ranging from 136009 to 152365), and did not show a clear trend in relation to the NIR treatment time. This was correlated to the non-significantly different viable spore numbers in these samples (shown Figure 3). However, the sample treated with SWCNTs alone did not show the highest level of DPA release compared to all SWCNTs-NIR treated samples, although the viable spore number in SWCNTs-treated sample was significantly higher than those in SWCNTs-NIR treated samples (shown in Figure 3). The possible reason for this result was that the SWCNTs-NIR treatment stimulated the germination and increased DPA release from the surviving spores which compensated the effect of less viable spores in the SWCNTs-NIR treated samples.

### Alterations in the expression levels of germination, regulation and virulence related genes in *B. anthracis* spores treated with SWCNTs-NIR

The expressions of germination-related genes are essential for the germination step. Spore germination has been studied intensively. Recently, 31 genes required for *B. anthracis* sporulation and germination were identified [34]. A few studies have been reported on how the expression of sporulation and germination related genes in *B. anthracis* spores are affected under different physical intervention techniques, such as heat treatment, pasteurization, and microfiltration [35]. Spore germination process is initiated by germinants, which are believed to bind to germination (Ger) receptors in most cases [36,37]. The Ger receptor genes are organized mostly as tricistronic operons that encode three distinct membrane-associated proteins [37,38]. In *B. anthracis*, genome sequencing revealed six genomic (*gerA*, *gerH*, *gerK*, *gerL*, *gerS*, and *gerY*) and one plasmidic (*gerX*) *ger* receptor operons [39]. Gene *gerN* (BA1639) encodes germination protein GerN which is a homolog of a widely distributed family of cation transporters, and is required for spore germination with inosine. Among these germination related genes, we selected six genes to examine their expression levels, including *ger HC*, *gerKB*, *gerLA*, *gerLB*, *ger YA*, and *gerN*.

In order to examine the effect of SWCNTs-NIR treatment on *B. anthracis* spores at the molecular level, real-time PCR was used to test the expression levels of six germination-related genes identified by the transposon site hybridization (TraSH) assay [34], seven virulence-related genes and three regulatory genes. Table 1 shows the fold changes in the expression levels of all the detected genes in *B. anthracis* spores upon SWCNTs-NIR treatment in comparison to the untreated spores.

**Table 1:** **Fold changes of gene expression after different treatments on *B. anthracis *Sterne spores**

	**Gene**	**Control**	**SWCNTs-NIR 5 min**		**SWCNTs-NIR 25 min**	
		ΔCt^*^	**ΔCt**	Fold change^**^	**ΔCt**	**Fold change**
Germination related genes	*gerLA*	20.50 ± 0.84	20.31 ± 0.96	1.14	19.45 ± 0.71	2.07
	*gerLB*	22.61 ± 1.04	22.60 ± 0.99	1.01	21.54 ± 0.42	2.11
	*gerYA*	19.80 ± 0.78	20.58 ± 0.27	0.58	20.83 ± 0.30	0.49
	*gerN*	21.74 ± 1.32	19.51 ± 0.91	4.68	21.31 ± 0.33	1.35
	*gerHC*	20.84 ± 1.14	20.64 ± 0.54	1.14	20.61 ± 1.01	1.17
	*gerKB*	21.27 ± 1.12	20.50 ± 0.92	1.71	23.17 ± 3.60	0.26
Regulatory genes	*atxA*	22.48 ± 0.39	23.36 ± 0.47	0.54	23.64 ± 0.66	0.45
	*pagR*	21.85 ± 0.18	23.15 ± 0.46	0.41	22.61 ± 0.82	0.59
	GBAA1941	22.36 ± 0.30	20.75 ± 0.80	3.05	21.39 ± 0.14	1.96
Virulence related genes	*cya*	21.91 ± 0.15	23.02 ± 0.13	0.46	23.79 ± 0.41	0.27
	*lef*	22.74 ± 0.33	23.38 ± 0.14	0.64	24.74 ± 0.18	0.25
	*pagA*	21.45 ± 0.52	22.35 ± 0.24	0.54	23.07 ± 0.34	0.33
	GBAA5564	22.59 ± 0.34	22.14 ± 0.17	1.37	22.75 ± 0.22	0.89
	GBAA0797	22.62 ± 0.13	22.83 ± 0.20	0.86	22.63 ± 0.30	0.99
	*purF*	23.51 ± 0.74	24.92 ± 0.19	0.37	23.23 ± 0.23	1.21
	*srtB*	23.78 ± 0.06	26.13 ± 0.89	0.2	24.30 ± 0.10	0.7

*The ΔCt value (Ct, threshold cycle) of each tested gene was a relative expression level compared to the value of housekeeping gene 16S rRNA*.* The ΔCt values are expressed as the average of three replicates ± standard deviation.

**The fold change is determined as 2^-ΔΔCt^.

Upon the treatment of 20 μg/mL SWCNTs coupled with different time of NIR treatment, the expression levels of the six tested germination-related genes were changed with various folds, and the results were related to the NIR treatment time. The expression level of *gerHC* stayed the same compared to that of the untreated samples regardless of the NIR treatment time. The *gerKB* gene increased its expression level to 1.7 fold upon SWCNTs with 5 min NIR treatment, and decreased to the value of 0.26 fold of the control samples upon SWCNTs with 25 min NIR treatment. GerK acts cooperatively with GerB nutrient receptor to trigger germination with L-asparagine plus a mixture of glucose, fructose, and K + (GFK) and also with L-alanine plus GFK [29,38]. These results suggested that in the presence of SWCNTs, shorter time NIR treatment (5 min) slightly activated the *gerKB* expression, but longer NIR treatment (25 min) inhibited its expression. *gerN* exhibited the similar trend to *gerKB* but with higher magnitude of changes, its expression level increased 4.7 fold upon SWCNTs with 5 min NIR treatment, and returned to 1.4 fold upon 25 min NIR treatment. *gerLA* and *gerLB* showed the same alteration trend that their expression levels almost did not change upon SWCNTs with 5 min NIR treatment but increased by 2 fold upon SWCNTs with 25 min NIR treatment. In *B. anthracis*, the GerL and GerK receptors are jointly required for the alanine germination pathway [40]. *gerYA* was the only one out of the six tested germination-related genes that decreased with NIR treatment. The *gerY* operon was found to be dispensable for recognition of all known germinant molecules in *B. anthracis*[40], but the role of GerY in *B. anthracis* spore germination is not clarified.

The detailed mechanisms of why the expression levels of some of the germination-related genes were increased, while some of them were decreases or stayed the same is not clear. -These molecular level changes might affect the germination of spores (including the release of DPA) and the lag phases in their growths for those SWCNTs-treated samples which we observed.

Besides the germination-related genes, we also examined the expression levels of three regulatory genes, *atxA*, *pag R*, GBAA1941, and seven virulence-related genes, *cya*, *lef*, *pagA*, *purF*, *srtB*, BGAA0797, and GBAA5564, upon SWCNTs-NIR treatment. These genes are important during the early infection of *B. anthracis*. Among the three regulatory genes, AtxA was isolated as a toxin gene activator 15 years ago and shown to be a *B. anthracis* master regulator [41]. AtxA controls the expression of more than a hundred genes belonging to all genetic elements, the chromosome and both virulence plasmids, including those encoding the major virulence factors [41]. AtxA is a transcriptional activator that controls the expression of three toxin genes, including the genes *pagA*, *cya*, and *lef* which encode the toxin component protective antigen (PA), edema factor (EF), and lethal factor (LF), respectively. PagR is a transcriptional repressor in *B. anthracis* that controls the chromosomal S-layer genes *eag* and *sap*, and down regulates the *pagA* gene by direct binding to their promoter regions [42]. GBAA1941 gene is a putative regulatory gene and its product shares significant homology with a family of transcriptional regulators (MarR) which includes proteins critical for control of virulence factor production, bacterial response to antibiotic and oxidative stresses and catabolism of environmental aromatic compounds [43]. GBAA1941 locus is important for *B. anthracis* pathogenesis [44]. As shown in Table 1, upon SWCNTs-NIR treatment, the expression levels of *atxA* and *pagR* decreased to about half compared to that of the untreated samples regardless of the NIR treatment time, while GBAA1941 gene increased its expression level by 3.05-and 1.96-fold at the NIR time 5 min and 25 min, respectively. The results suggested that SWCNTs-NIR treatment caused alterations in regulatory genes in *B. anthracis* spores, but the alteration levels varied in different regulatory genes depending on the functions of these genes in relation to the treatment.

In view of the expression levels of virulence genes, three toxin genes, *cya*, *lef*, and *pagA*, which are regulated by *atxA* gene decreased their expression levels accordantly to 0.46 -0.64 folds upon SWCNTs with 5 min NIR treatment, and to 0.25-0.33 folds upon 25 min NIR treatment. The alteration in the expression level of *pagA* after treatments was also correlated to the down regulation of the *pagR* gene. Gene GBAA5564 encodes a conserved hypothetical protein usually highly expressed during early infection [44]. Gene GBAA0797 encodes permease protein of the ABC transporter. The SWCNTs-NIR treatments on spores did not show significant effects on the expression levels of either GBAA5564 or GBAA0797 (varying from 0.86 to 1.37 folds). Gene *srtB* encodes sortase which is an enzyme possibly critical in the early stages of inhalation anthrax [45]. The disruption of *srtB* had no effect on the ability of the bacteria to grow in rich culture media, but mutations in this gene dramatically attenuated growth of the bacterium in J774A.1 cells [45]. Gene *purF* encodes amidophosphoribosyltransferase which is an enzyme in purine nucleotide biosynthetic process. Upon SWCNTs-NIR treatment, 5 min treatment showed significant inhibition effect on *srtB* expression in *B. anthracis* spores, decreasing the expression level to 0.2 fold (5 min) of the control samples. The SWCNTs-NIR 5 min treatment also significantly inhibited *purF* expression to 0.37 fold of the control samples, but 25 min treatment did not show obvious effects on this gene (1.21 fold).

In summary, SWCNTs-NIR treatments significantly decreased the expression levels of 3 out of 7 detected virulence-related genes and 1 out of 3 detected regulatory genes when the NIR time was 5 or 25 min. No obvious increase was observed in any of the tested virulence-related genes. However, only a very small number of genes were examined in this study.

## Conclusions

We have investigated the effect of SWCNTs-NIR treatment on *B. anthracis* spores. At 10 μg/mL, SWCNTs alone only exhibited little sporicidal effect on *B. anthracis* spores, while SWCNTs coupled with 20 min NIR significantly improved the antimicrobial effect by doubling the percentage of viable spore number reduction compared with SWCNTs alone treatment (88% vs. 42%). The SWCNTs-NIR treated samples showed slightly more rapid growth, and released similar level of DPA during germination despite the less viable spores in them. The results suggested the dual effect of SWCNTs-NIR treatment on *B. anthracis* spores, which enhanced the sporicidal effect and stimulated the germination of surviving spores. Molecular level examination showed that SWCNTs-NIR treatment altered the expression of germination, regulatory, and virulence-related genes in *B. anthracis.* The alterations in germination-related genes were correlated with the stimulated germination of surviving spores (including the release of DPA).

## Materials and methods

### SWCNTs

Suspensions of SWCNTs (with surface group -OH) in deionized (DI) water with varied length of 1-5 μm at concentration of 1 mg/mL were purchased from Nanolab, Inc. (Newton, MA). According to the manufacturer report, SWCNTs were produced using a chemical vapor deposition process with high yield and purity, with little or no amorphous carbon. The data of the energy-dispersive X-ray spectroscopy analysis conducted by means of SEM (SEM-EDX) provided by the manufacturer indicated that the SWCNTs contain 95.93% weight percentage of carbon and 4.07% of other elements (Na, Al, Si, S, and Fe). Based on the SEM and TEM images of SWCNTs [5], the products presented clusters in the nanotube bundles and terminals which most likely were the catalyst metals used in SWCNTs synthesis [46]. The presence of catalyst clusters in SWCNTs was observed in products used in studies by other researchers [46,47], but low concentrations of residual catalysts in CNT products were uncorrelated to CNTs’ toxicity to bacterial cells [3,4,48,49].

### *B. anthracis* spore preparations

*B. anthracis* spores were prepared and purified using the similar procedure as previous reported [26]. One colony of *B. anthracis* Sterne 34 F2 (Colorado Serum Company, Denver, CO) from a Luria-Bertani (LB) agar plate was used to inoculate 25 mL Disfco Sporulation Medium (DSM). The inoculum was incubated in a shaker incubator at 37°C with agitation of 225 rpm until mid-log phase, followed by adding 225 mL of pre-warmed (37°C) fresh DSM. The culture was incubated in the shaker incubator until > 95% of the culture were free spores. To purify the spores, the spore culture was first heated at 70°C for 20 min to kill the remaining vegetative cells, and then centrifuged at 8,000 × g at 4°C for 10 min. The pellet was washed with cold (4°C) DI water eight times to remove cell debris. The purified spores were suspended in 4°C DI water and stored at 4°C. Before each experiment, desired volume of the stock spores were washed with 4°C DI water twice for further use.

### SWCNTs and NIR laser treatment on *B. anthracis* spores

Aliquots of 150 μL *B. anthracis* spore suspension in DI water with concentrations of ~10^5^ to 10^6^ cfu/mL were introduced into 1.5 mL eppendorf centrifuge tubes. The samples were added with desired volumes (2 to 20 μL) of SWCNTs, and the total volume of each sample was adjusted to 200 μL with DI H_2_O with the final concentration of SWCNTs in the samples varying from 10 μg/mL to 100 μg/mL. The samples were mixed with constant rotation at 15 rpm on a rotation mixer (Model: RKDDYNAL) from Dynal Biotech, Inc. (Lake Success, NY) at room temperature for 30 min, followed by transferring the suspensions into quartz cuvettes (Fisher Scientific, Pittsburgh, PA) for NIR laser treatment. For NIR treatment, we used the same experiment setup as our previous study [20]. The Tsunami mode-locked Ti:Sapphire laser system (Newport Spectra-Physics) was used, which was pumped by the Millennia (Newport Spectra-Physics) diode pumped solid state 532 nm 15 W continuous wave laser. The resulting mode-locked Ti:sapphire laser was tunable from 700 nm to 1080 nm. An optical lens was used to focus the laser beam on the quartz cuvette in which the spore suspension with SWCNTs was placed. Continuous NIR light at 800 nm wavelength with output power of 360-410 mW was used to treat the samples for 5 to 30 min. The laser beam spot was approximately 2 mm in diameter. The laser density was in the range of 1.15-1.31 mW/cm^2^. A thermocouple probe was inserted into the suspensions to monitor temperature change in the bulk solution during NIR treatment.

### Determination of viable spore number and measurement of spore outgrowth

After SWCNTs or SWCNTs-NIR treatment, the viable spore number in the treated samples was determined using the traditional plating method. The treated samples were serially (1:10) diluted with DI water. 100 μL of appropriate dilutions were surface plated on LB agar plates in triplicates. Colonies were counted after 24 h of incubation of the plates at 37°C. The viable spore number was calculated as colonies forming units per mL (cfu/mL).

The outgrowths of the treated spores were examined by optical density (OD) measurement at 600 nm. 100 μL of spore suspensions were taken from the 200 μL of treated samples and added to 5 mL Nutrient broth. The samples were then incubated in a shaker incubator at 225 rpm at 37°C for growth.

### Measurement of dipicolinic acid (DPA) release during spore germination

Spores (OD600 = 0.5) treated with SWCNTs or SWCNTs-NIR were germinated in a 96-well black plate with clear bottom at 37°C in 200 μL of germination solution which contained 25 mM HEPES buffer, 2 mM L-alanine, and 50 μM TbCl_3_. DPA release was monitored by real-time measurement of fluorescence signals at 545 nm with excitation at 270 nm, which are appropriate wavelengths for the Tb^3+^ -DPA complex using the Spectra Max M5 spectrophotometer (Molecular Devices, LLC, Sunnyvale, CA) [50,51]. For each sample, the background detected at time zero was used as the blank. The percentage of DPA release at a sample time was calculated by dividing the value of relative fluorescence units (RFU) at that time by the maximum value of RFU at 1 h of germination.

### RNA isolation and cDNA synthesis

RNA isolations were performed immediately after SWCNTs or SWCNTs-NIR treatment. Treated spores were collected by centrifugation at 8,000 × g at 4°C for 5 min, and washed twice with 0.5% Tween 20 solution. Spores were centrifuged and resuspended in RNAwiz buffer from RiboPure-Bacteria kit (Life Technologies, Carlsbad, CA). Zirconia beads were added and vortexed for 10 min, followed by a 5-min break. This vortexing-break step was repeated 5 times to ensure the disruption of the spores. RNA was purified using RiboPure-Bacteria kit according to the manufacturer’s protocol. RNA quality and quantity were measured using NanoDrop 2000 spectrophotometer (NanoDrop Technologies Inc., Wilmington, DE). RNA samples were then treated with DNase I from the RiboPure-Bacteria kit according to the manufacturer’s instruction. cDNA was synthesized using the SuperScript III First-Strand Synthesis SuperMix kit (Life Technologies, Carlsbad, CA) with random primers following the manufacturer’s protocol.

### Primer design and specificity test

In order to examine the effect of SWCNTs-NIR treatment on *B. anthracis* spores at the molecular level, we examined the expression levels of ten regulation and virulence-related genes and six germination related genes in *B. anthracis* Sterne. The gene sequences of the regulation and virulence- related genes were acquired from NCBI website (http://www.ncbi.nlm.nih.gov) and specific primers were designed for the analysis of transcriptional gene expression using real-time PCR (Table 2). The primer sequences for the amplification of six germination-related genes and 16S rRNA gene were acquired from a previous publication [35]. The specificities of all the primers designed in this study were tested by Blast function from Primer-Blast and further confirmed by colony PCR. Primers were synthesized and purchased from Eurofins MWG Operon (http://www.operon.com). To run the colony PCR, a single colony of *B. anthracis* Sterne on LB agar was picked and suspended in 100 μL sterilized DI-H_2_O in a 1.5 μL centrifuge tube. The colony suspension was heated in a water bath with boiling water for 5 min, followed by incubation at -80°C for 10 min. This step was repeated 3 times, and then centrifuged at 15,000 × g for 3 min. The supernatant was used as template DNA for PCR amplification. The PCR reaction system contained: 2 μL of 10 × PCR buffer containing Mg^2+^, 2 μL of 2 mM dNTP mix, 0.4 μL of each 10 μM forward and reverse primer, 0.5 unit of Taq DNA polymerase (Thermo Fisher Scientific Inc, Foster City, CA), 2 μL of DNA template, and DI water to a final volume of 20 μL. The PCRs were performed in an Eppendorf Mastercycler (Eppendorf Scientific, Inc., Westbury, NY) under the following conditions: 94°C for 5 min; 32 cycles of 93°C for 30 s, 57°C for 40 s, and 72°C for 40 s; and finally 72°C for 5 min. The presence of the PCR product was confirmed by electrophoresis on 2.0% agarose gels and staining with ethidium bromide.

**Table 2:** Primer sequences designed in this study for the detection of specific genes through real-time PCR

**Gene**	**Primer sequence**	**Product size (bp)**	**Description**
atxA	CGGACTGTTTTATCTGCGACCTGT	127	transcriptional activator AtxA
	CACCGATATCCATCGAAAAGGAACA		
cya	TCGGATTGGGGCCCTGTAGC	80	calmodulin-sensitive adenylate cyclase
	TCGACAGCTAATTGTTGACCATGC		
lef	TCTTGCCAGCATCCGTTGATCT	129	lethal factor
	ATCGAGCCACAGCATCGTGA		
pagA	GGGTGGATACAGGCTCGAACTGG	115	protective antigen
	ACCGCCGCTATCCGCCTTTC		
pagR	ACAATGGCACATCCTATGCGTT	116	transcriptional repressor
	TGCTGGGATACAGTTGATTGTGGT		
purF	GAACTGCGCGAGCAAGGGGT	137	Amidophosphoribosyl-transferase
	GCCTCGCGAAGCATGCGAAC		
srtB	TGCTGCAGCGCTTTGAACTGT	96	sortase B
	AGTAATGGCCGAAGCACAAAACA		
GBAA1941	ACACAAGGCGGTATTTCTCGAATGT	144	MarR family transcriptional regulator
	TGCAAGTTGCTCTGGCAATGCT		
GBAA5564	TGGTTGGTTCGGCGGTTATGGT	82	hypothetical protein
	TGCCATGAATACACCAGGGCG		
GBAA0797	CCACGCGTTGCACCAAGAGC	130	ABC transporter permease
	TGGTATTCGGTGGTATTGCCGCT		

### Real-time PCR and data analysis

Real-time PCRs were performed in 96-well plates on a 7500-Fast real-Time PCR system (Applied Biosystems, Foster City, CA, U.S.A.). Each well contained 20 μL reaction mixtures, including 10 μL of 2× Fast SYBR® Green Mster Mix (Applied Biosystems, Forster City, CA, U.S.A.), 0.4 μL of each 10 μM forward and reverse primer, 0.7 μL cDNA, and 7.8 μL DI-H_2_O. Each sample was assayed in triplicates and the average value was used for data analysis. Amplification condition involved an initial denaturation at 95°C 10 min and 40 cycles of 95°C 15 s and 60°C 1 min. Fluorescence data were collected at the annealing step. A final dissociation step was performed to verity amplification specificity. Results were analyzed using 7500 Fast System SDS software v 1.4 (Applied Biosystems, Forster City, CA, U.S.A.) provided with the thermocycler. The ΔCt value (Ct, threshold cycle) of each tested gene was a relative expression level compared to the expression of housekeeping gene 16S rRNA. The fold changes of transcript abundance between the treated sample and the untreated control were calculated using the formula 2^-ΔΔCT^[52].

## Abbreviations

CNTs: Carbon nanotubes; SWCNTs: Single walled carbon nanotubes; NIR: Near infrared; PCR: Polymerase chain reaction; DPA: Dipicolinic acid; RFU: Relative fluorescence units; UV: Ultraviolet; VIS: Visible.

## Competing interests

The authors declare that they have no competing interests.

## Authors’ contributions

XD and YT developed the methods and performed the experiments. MW and BV provided instruments and helped to conduct the experiments. LY designed the study and supervised the work. XD and LY wrote and revised the manuscript. All authors read and approved the final manuscript.
